# *Humulus lupulus* Promoting Osteoblast Activity and Bone Integrity: Effects and Mechanisms

**DOI:** 10.3390/biology14050582

**Published:** 2025-05-21

**Authors:** Nahuel Ezequiel Wanionok, Germán Andrés Colareda, Juan Manuel Fernandez

**Affiliations:** 1 Laboratorio de Investigaciones en Osteopatías y Metabolismo Mineral (LIOMM), Facultad de Ciencias Exactas, Universidad Nacional de La Plata (UNLP), Centro de Investigaciones Científicas (CIC), La Plata B1900, Argentina; jmfernandez@biol.unlp.edu.ar; 2 Farmacología-GFEYEC, Departamento de Ciencias Biológicas, Facultad de Ciencias Exactas, Universidad Nacional de La Plata (UNLP), La Plata B1900, Argentina; gcolareda@biol.unlp.edu.ar

**Keywords:** osteoblast, bone marrow stem cells, bone microarchitecture, biomechanics, hops, phytoestrogens

## Abstract

Osteoporosis is characterized by an imbalance between bone formation and resorption, leading to decreased bone mass and an increased fracture risk, mainly associated with aging. Current treatments include anti-resorptive and anabolic drugs. However, these often have side effects, leading many patients to seek natural biological alternatives. We have demonstrated previously that hops extract, rich in compounds with estrogenic activity classified as phytoestrogens, exerts osteogenic effects by promoting the osteoblastic differentiation of bone marrow stem cells (BMSCs) while inhibiting osteoclast activity *in vitro*. In our study, young male Sprague Dawley rats were randomized into two groups: one received hops extract (LPL, 1% *w*/*v* in drinking water) for two months, and the other drank water alone (C). The rats were euthanized, and their femurs were dissected and processed for static histomorphometry and bone biomechanics. Additionally, BMSCs were isolated from the humeri to evaluate their osteogenic potential. Our result demonstrated that LPL treatment enhanced the osteogenic potential of humeral BMSCs in *ex vivo* assays, upregulating osteogenic genes and downregulating pro-resorptive markers. These findings correlated with improved femoral bone microarchitecture and biomechanical parameters. In conclusion, a two-month treatment with LPL enhanced the osteogenic capacity of BMSCs, improving bone microarchitecture and biomechanical properties. These results suggest its potential as a natural alternative for promoting bone health.

## Simple Summary

Osteoporosis is the most prevalent metabolic bone disease, with its incidence increasing over time. This condition, characterized by an imbalance between bone formation and resorption, poses a significant public health challenge marked by increased fracture risk. Moreover, adherence to current treatments is often limited due to their potential side effects. In this study, we investigated the osteogenic potential of a hops extract in Sprague Dawley rats. Over two months, the rats were administered a 1% *w*/*v* hops extract, which contains substances with estrogenic activity for bone health, while a control group received water. The outcomes were assessed through static bone histomorphometry and evaluation of bone biomechanics following euthanasia. Our study shows that hops extract helps stem cells from the bone marrow to develop into bone-forming cells more effectively. It increases the activity of genes that build bone and decreases the activity of those that break it down. As a result, bones become stronger and healthier in structure. This suggests that the hops extract may help slow down bone loss and support the growth of new bone. These findings indicate that hops extract could be a helpful natural supplement for preventing or managing bone diseases such as osteoporosis.

## 1. Introduction

Osteoporosis (OP) is a disease characterized by bone tissue loss and an increased risk of fragility fractures. It is the most common metabolic bone disease, in which bone macro- and microarchitecture deteriorate through an imbalance between bone formation and resorption [1]. Although OP largely affects postmenopausal women, it also has implications for aging men. Due to the increase in global life expectancy and population growth, the incidence of OP is steadily increasing, which will have a great impact both clinically and economically [2]. Moreover, as a chronic condition, OP requires long-term treatment with high adherence, making the management of side effects particularly important. Consequently, an increasing number of patients are turning to natural-product-based therapies as complementary or alternative treatment options [3].

As previously mentioned, OP results from an imbalance in bone remodeling. Bone marrow stem cells (BMSCs) differentiate into various cell types, including osteoblasts, which are responsible for bone tissue synthesis. This process begins with the production of collagen and then goes through its mineralization, primarily mediated by alkaline phosphatase. As for molecular mechanisms, several signaling molecules regulate osteoblastic proliferation and differentiation, including Runt-related transcription factor 2 (Runx2), which is expressed by mesenchymal cells from the earliest stages of skeleton formation and is the principal transcriptional regulator required for the development of the osteoblastic lineage [4]. On the other hand, osteoblasts regulate osteoclastic activity to maintain bone mass. Osteoclastogenesis is initiated through cell–cell interaction between the receptor activator of NFκB ligand (RANKL), expressed by osteoblasts and osteocytes, and its receptor RANK, expressed by pre-osteoclasts of hematological lineage. An increase in RANKL expression promotes osteoclast maturation and, consequently, bone resorption. Additionally, osteoblasts secrete osteoprotegerin (OPG), a protein that binds to RANKL with approximately 500-fold higher affinity than RANK [5], leading to the inhibition of osteoclastogenesis. Thus, the development of OP is primarily established by an imbalance between osteoblastic differentiation from BMSCs and osteoclastogenesis modulated by the RANKL/OPG ratio [6].

In postmenopausal women, the main mechanism leading to OP is estrogen deficiency due to ovarian decline. In this context, estrogens are widely used as treatments to mitigate the onset of OP. These hormones are characterized by promoting osteoblastogenesis and reducing bone resorption, thereby performing an anabolic effect on bone tissue. Their main action is to inhibit the differentiation and activity of osteoclasts through the modulation of signals from osteoblasts, specifically a decrease in osteoclast-stimulating cytokines (IL-1, IL-6, TNF-α) and an increase in the factors that antagonize resorption (IGF-1, TGF-β). Additionally, estrogens increase the production of OPG by osteoblasts and promote their bone-forming activity since they increase the synthesis of bone matrix components while reducing osteocytic and osteoblastic apoptosis [4,7,8]. In recent years, alternative treatments have been explored to reduce the adverse effects of exogenous estrogen, among which phytoestrogens stand out. These compounds show structural similarities to estrogens and share certain hormonal effects [9,10,11,12,13], and when administered, they promote beneficial effects on bone tissue, practically without exhibiting side effects.

Recently, hops have attracted significant interest in medical areas due to their potential benefits on health, including bone tissue. Lupulin is a gland found in the female inflorescences of hops, producing a variety of compounds, some of which are classified as phytoestrogens [3]. We previously investigated the effects of an ethanolic hop extract (LPL) added to a cell culture medium on the proliferation and osteogenic differentiation of BMSCs *in vitro* [14]. After evaluating different concentrations, we identified 100 ng/mL as the most favorable concentration for promoting cell proliferation. Moreover, BMSCs treated with LPL exhibited increased alkaline phosphatase activity, type I collagen production, and extracellular matrix mineralization. In parallel, the upregulation of osteogenic genes such as Runx2 and osteocalcin was observed. Additionally, a reduction in the RANKL/OPG ratio suggested a reduced capacity of osteoblasts to promote osteoclastogenesis in the presence of hops extract.

Based on these recent promising results, we hypothesized that hop extract treatment may exert beneficial effects on bone microarchitecture and biomechanical properties through oral administration. This could support the development of preventive or alternative treatments for conditions such as OP.

## 2. Materials and Methods

### 2.1. Animal Care and Management

We used Sprague Dawley young male rats bred in-house at Farmacología-GFEYEC, Faculty of Exact Sciences, National University of La Plata, Argentina. The rats were housed in pairs in cages with wood-shaving bedding and standard rodent laboratory chow. The housing room was maintained in a 12:12 h light/dark cycle and 23 ± 3 °C with humidity at 30–70%. The experimental animal protocol was approved by the Institutional Ethics Committee of the Facultad de Ciencias Exactas, UNLP (Protocol Number 006-45-23 approved in June 2024).

### 2.2. Hop Ethanol Extract Preparation

The plant material used in our experiments was provided by the brewing industry in the form of powdered hops (*Humulus lupulus* L., *Cannabaceae*) inflorescences (batch number 01, harvest 2019, 9.0% alpha-acids), cultivated in Río Negro Province, Argentina (41°58′ S, 71°32′ W). The hops powder was stored under appropriate conditions and vacuum-packed. A total of 10 g of hops powder was weighed and suspended in 100 mL of ethanol 70% for at least 48 h in an airtight container. During maceration, the mixture was shaken twice daily and stored in a sunlight-protected location at ambient temperature. Once the maceration process was complete, the mixture was filtered to obtain 100 mL of hop tincture. The tincture was prepared using the same plant material, by the same investigator, in the same laboratory, and under the same conditions as in a recent study, in which phenolic compounds (particularly flavonoids and phenolic acids) were found to be the main components. As previously described by Colareda et al., High-Performance Liquid Chromatography in a reverse-phase system (RP-HPLC-DAD) was used to characterize the tincture for the presence of flavonoids. The compounds were identified by comparing their retention times and UV spectra with those of the reference standards and data published in the literature. The main flavonoids were successfully identified, highlighting prenylated flavonoids such as xanthohumol (the most abundant), as well as β-acids including colupulone, cohulupone, and lupulone. Additionally, traces of flavones and isoflavones such as genistein, glycosylated flavones, and daidzein were detected [14,15].

The yield (%) of hop tincture (Weight of dry extract/Weight of hop inflorescence powder × 100) was 27.0 ± 0.5 %*w*/*w* gram of dry residues of extract per gram of hops (n = 2 determinations).

To prepare the hop tincture dilution in drinking water, the ethanol in the extract was first removed by evaporation at room temperature. Then, 10 mL of hop extract was mixed with water to reach a final volume of 100 mL, resulting in a final concentration of 1 % *w*/*v*.

### 2.3. Study Design

Ten clinically healthy male Sprague Dawley rats were randomized into two groups (age 5 months, body weight 419.8 ± 29.7 g).

Animals of the control group (C) received sterile water alone ad libitum. The other group (LPL) was treated with a hop extract added to the drinking water (1% *w*/*v*). These conditions were maintained for two months. Throughout the treatment, 28 determinations were made to calculate the ml of extract (LPL group) consumed during 24 h, and the ratio was calculated based on the body weight of animals (mL/kg/day) for each determination. Then, the daily administered dose (571.33 ± 39.7 mg/kg/day, n = 28) was estimated.

After the treatment period, non-fasting rats were weighed and anesthetized with an overdose of thiopental (40 mg/kg intraperitoneally) (Euthanyle ^®^, Brouwer, Buenos Aires, Argentina) after analgesia with tramadol (10–20 mg/kg subcutaneously). The animals were then euthanized by heart extirpation by opening the chest. Both femora were dissected, cleared of soft tissue, and fixed in 10% phosphate-buffered formalin for 48 h. Subsequently, they were washed under running water, stored in 70% ethanol at 4 °C, and processed as described below. Additionally, the humeri were dissected and processed for the isolation of bone marrow stem cells (BMSCs).

### 2.4. Bone Structural Parameters

#### 2.4.1. Bone Histomorphometry

Each left femur was prepared for quantitative histomorphometric evaluation. The bones were decalcified using a 10% EDTA solution and, subsequently, they were embedded in paraffin. Sections of the proximal region that were 5 μm thick were obtained with a microtome (RMT-20 Type Erma, TechLabs, New Delhi, India) and stained with hematoxylin and eosin (H&E). Images were captured using a Nikon Coolpix 4500 digital camera mounted on a Nikon Eclipse E400 microscope (Nikon Corporation, Tokio, Japan) and analyzed with the ImageJ software (version 1.54n). The bone microarchitecture of proximal secondary spongiosa was analyzed: trabecular bone area (as %), adiposity in bone marrow (as adipocytes/mm^2^), and trabecular bone osteocytic density (as osteocytes/mm^2^) [16,17].

#### 2.4.2. Three-Point Bending Test

The biomechanical properties of each right femur were assessed using a three-point bending test with a Digimess TC500 electromechanical testing machine. The test employed a 500 N load cell (Interface, Scottsdale, AZ, USA) at ambient temperature, with a 20 mm support span and a constant loading speed of 5 mm/s. Load (F), applied in the anteroposterior axis, and displacement (D) up to the point of fracture were recorded. These data were used to generate a stress–strain curve. The maximum force endured before fracture (FMax) was taken as an indicator of ultimate bone strength. Stress–strain curves were used to derive the mechanical properties of the whole bone. From these curves, maximum elastic displacement (Dy) at the yield point and the corresponding yield load (Fy) were identified. Bone stiffness at the yield point was calculated as the ratio Fy/Dy. Energy absorbed by the bone during deformation, referred to as work-to-fracture, was determined from the area under the stress–strain curve. This absorbed energy (Eabs) was further divided into contributions from the elastic (E-Eabs), plastic (P-Eabs), and total deformation (T-Eabs) phases [17].

### 2.5. Ex Vivo Cell Cultures

#### 2.5.1. Bone Marrow Stem Cell Isolation and Maintenance

BMSCs were isolated from the humeri of all the experimental animals. After sectioning both epiphyses, the diaphyseal cavity was flushed with Dulbecco’s Modified Eagle Medium (DMEM) (Invitrogen, Buenos Aires, Argentina) under sterile conditions to collect the bone marrow. Cells were cultured in DMEM supplemented with 10% fetal bovine serum (FBS) (Natocor, Córdoba, Argentina), 100 U/mL penicillin, and 100 μg/mL streptomycin and maintained at 37 °C in a humidified incubator with 5% CO_2_ and 95% air [18]. After 24 h, non-adherent cells were removed by changing the culture medium. Adherent cells were expanded with periodic medium changes two to three times per week. Once confluence was achieved, cells were resuspended using trypsin/EDTA and replated.

#### 2.5.2. BMSCs’ Osteogenic Differentiation

BMSCs were seeded into 24-well plates and maintained in DMEM supplemented with 10% FBS at 37 °C with 5% CO_2_. At 70% cell confluence, osteogenic differentiation was initiated by culturing BMSCs for 14 days in an induction medium containing DMEM with 10% FBS, 25 μg/mL ascorbic acid, and 5 mM sodium β-glycerophosphate [18]. Osteoblastic differentiation was evaluated through the expression of type I collagen and the enzymatic activity of alkaline phosphatase (ALP).

After the 14-day differentiation period, cells were rinsed with phosphate-buffered saline (PBS) and lysed in 250 μL of 0.1% Triton X-100. An aliquot of the lysate was used to quantify ALP activity by assessing the hydrolysis of p-nitrophenyl phosphate (p-NPP) into p-nitrophenol (p-NP) at 37 °C, with absorbance measured at 405 nm [18]. Another aliquot was analyzed for total protein content using Bradford’s technique [19]. For the evaluation of type I collagen production, cells were fixed with Bouin’s solution for one hour, rinsed, and stained with Sirius Red. The stained cells were homogenized with 0.1 N sodium hydroxide, and absorbance was measured at 550 nm [18].

#### 2.5.3. Gene Expression of Bone Marrow Stromal Cell Osteogenic and Pro/Anti-Resorptive Markers

The gene expression analysis of osteogenic and bone remodeling-related markers in BMSCs was evaluated using reverse transcriptase polymerase chain reaction (RT-PCR). After 14 days of osteogenic differentiation, total RNA was isolated from the cells with TRIZOL reagent, following the protocol provided by the manufacturer (Invitrogen Life Technologies, Argentina). Semi-quantitative RT-PCR was carried out to evaluate the expression of alkaline phosphatase (ALP), type I collagen, Runx2, osteocalcin (Oc), receptor activator of nuclear factor-κB ligand (RANKL), and osteoprotegerin (OPG), employing Moloney Murine Leukemia Virus Reverse Transcriptase (MMLV-RT) (Invitrogen, Argentina). The housekeeping gene β-actin was used to normalize the gene expression of all of the markers. Primers specific to each gene target were designed based on sequences obtained from the NCBI database using CLC Genomics Workbench (QIAGEN) and synthesized by Macrogen (Seoul, Republic of Korea) (Table 1). RT-PCR products were separated on agarose gels stained with GelRed, and band intensities corresponding to the expected sizes were quantified using ImageJ software. The ratio of RANKL/OPG expression was then calculated.

### 2.6. Statistical Analysis

The results are expressed as the mean ± SEM. Differences for each investigated parameter between the control and LPL groups were assessed by Student’s *t*-test using GraphPad InStat version 3.00 (GraphPad Software, Boston, MA, USA). *p* < 0.05 was considered significant for all statistical analyses.

## 3. Results

### 3.1. General Observations

No significant differences were observed in the body weight of the groups at the end of the study (430.7 ± 33.42 and 497.5 ± 79.2 g for LPL and control groups, respectively).

### 3.2. Bone Histomorphometry

**Figure 1:**
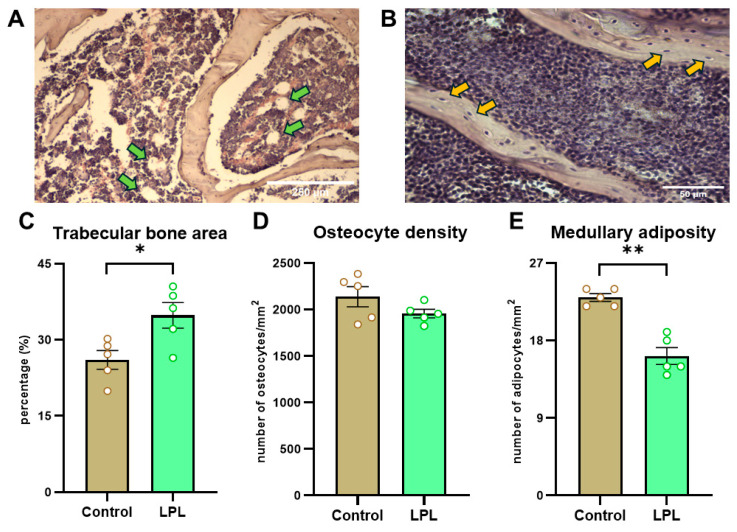
**Representative photographs of histologic sections from proximal metaphysis of femur rats and their quantification.** (**A**) Representative histologic section of trabecular bone tissue. Obj.: 10× (green arrow: adipocyte in bone marrow); (**B**) representative histologic section of trabecular bone. Obj.: 40× (orange arrow: osteocyte in trabeculae); (**C**) trabecular bone area (as % of total area); (**D**) trabecular bone osteocytic density (as number of osteocytes/mm^2^); and (**E**) medullary adiposity (as number of adipocytes/mm^2^). Results are expressed as the mean ± SEM. Differences: * *p* < 0.05, ** *p* < 0.01 vs. control.

Interestingly, we observed that treatment with LPL increased the relative trabecular bone area compared to the animals in the control group. Furthermore, this treatment appeared to prevent bone marrow adiposity, as a higher number of adipocytes per unit area was found in the control animals. However, no significant differences were observed in terms of osteocyte density within the trabecular bone of the femur. Figure 1 includes representative photomicrographs of the proximal femur metaphysis from rats of the different groups.

### 3.3. Three-Point Bending Tests

In line with the bone histomorphometric data, we demonstrated a significant increase in stiffness, yield point, maximum load, and work to fracture in the femora of rats treated with LPL compared to the control group (Table 2). However, no changes were observed in the energy absorbed in the elastic and plastic regions.

### 3.4. Osteogenic Potential of Bone Marrow Stem Cells (BMSCs)

**Figure 2:**
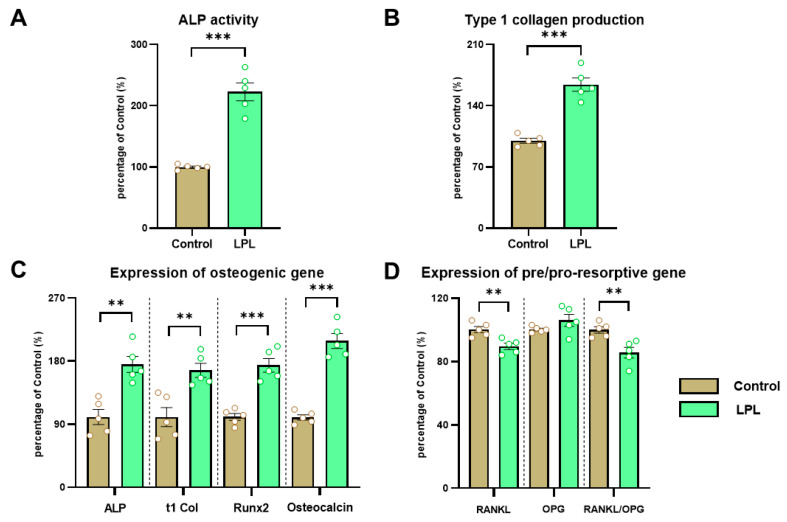
***Ex vivo* effect of oral LPL treatment on the osteogenic differentiation potential of BMSCs. BMSCs were evaluated for the following:** (**A**) alkaline phosphatase activity; (**B**) type I collagen production; (**C**) expression of osteogenic genes (ALP: alkaline phosphatase, t1 Col: type I collagen, Runx2: runt-related transcription factor 2); (**D**) expression of genes regulating osteoclastogenesis (RANKL: receptor activator of nuclear factor-κB-ligand; OPG: osteoprotegerin). Results are expressed as the mean ± SEM. Differences: ** *p* < 0.01, *** *p* < 0.001 vs. control.

An increase in the osteogenic capacity of the BMSCs obtained from animals treated with LPL was observed compared to the control group. Both ALP activity (Figure 2A) and type I collagen production (Figure 2B) were significantly higher as a result of the LPL treatment after 14 days of osteogenic differentiation.

### 3.5. Gene Expression of Osteoblastic and Pro/Anti-Resorptive Markers

The LPL treatment led to an *ex vivo* increase (versus control) in BMSCs’ gene expression of osteoblastic markers Runx2 and Oc (after 14 days of osteogenic differentiation). Additionally, an upregulation of type I collagen and ALP expression was observed, consistent with the results for BMSCs’ osteogenic capacity (Figure 2C).

In addition, RANKL expression was lower in BMSCs derived from animals in the LPL group, as was the RANKL/OPG ratio (Figure 2D). This suggests a reduction in the pro-resorptive profile of BMSCs.

## 4. Discussion

In recent years, studies have examined the effects of hops extract components such as 8-prenylnaringenin (8-PN), 6-prenylnaringenin (6-PN), xanthohumol (Xan), and isoxanthohumol (IXan) on bone tissue health. Their strong interaction with estrogen receptors, mainly with the alpha receptor present in bone cells, enables them to modulate bone formation and resorption pathways [3,20]. However, when used in high doses and for prolonged periods of time, they have been associated with several adverse effects, such as an increase in uterine weight, vaginal epithelium proliferation, and uterine bleeding [20,21,22,23]. On the other hand, the use of a hops extract could be a potential alternative since it contains a variety of different phytoestrogens that act synergistically, producing additive effects, and can thus be used in low concentrations, decreasing side effects.

In our previous *in vitro* study, we investigated the effect of LPL on the osteogenic capacity of BMSCs [15]. We demonstrated that at a concentration of 100 ng/mL, the proliferation of these cells was maximized. In addition, their osteogenic potential was enhanced compared to control cells, as evidenced by increased ALP activity, type I collagen production, and the upregulation of genes such as Runx2 and osteocalcin. This was accompanied by a decrease in the adipogenic potential of BMSCs. Furthermore, we observed a lower RANKL/OPG expression ratio, suggesting a decreased capacity for osteoclast formation due to the presence of LPL in the culture medium. Notably, all these effects were mediated through the MAPK pathway. Based on these results, we decided to investigate the effect of LPL in an *in vivo* assay.

Our current study was based on a two-month treatment of young male Sprague Dawley rats with hops extract (LPL) in their drinking water. Based on our determinations, the hops dose was comparable to that reported in other studies [24,25,26,27]. The outcomes of our study provide evidence that the administration of hops extract significantly influences bone health parameters in Sprague Dawley rats. Our findings support the hypothesis that this extract enhances osteogenic potential, evidenced by the upregulation of key osteogenic genes such as Runx2, which is critical for osteoblast differentiation, and osteocalcin, another important marker of bone formation. Similarly, Xan, the most abundant compound in hop cones [12], can increase ALP activity and Runx2 expression in bone cells [28]. Furthermore, bitter acids have been shown to promote osteoblast differentiation and reduce their apoptosis [29].

Our present results also indicate that hops could inhibit osteoclastogenesis by a decrease in the RANKL/OPG ratio, suggesting a decreased differentiation of monocytes into osteoclasts. Interestingly, Xan inhibits the signaling cascades promoted by RANKL, modulating osteoclastogenesis [30]. Moreover, Xan can be biotransformed to 8-PN through hepatic metabolism or gut microflora, indicating that estrogenic effects should be present, and could explain the decrease in the RANKL/OPG ratio via the activation of estrogen receptor α (ER-α) [31]. In addition, a recent report has found that β-bitter acids stimulate osteoblast activity and suppress osteoclast function [32]. Estrogens have also been described to increase the synthesis of type I collagen and ALP by osteoblasts [7], in line with our present results (Figure 2).

**Figure 3:**
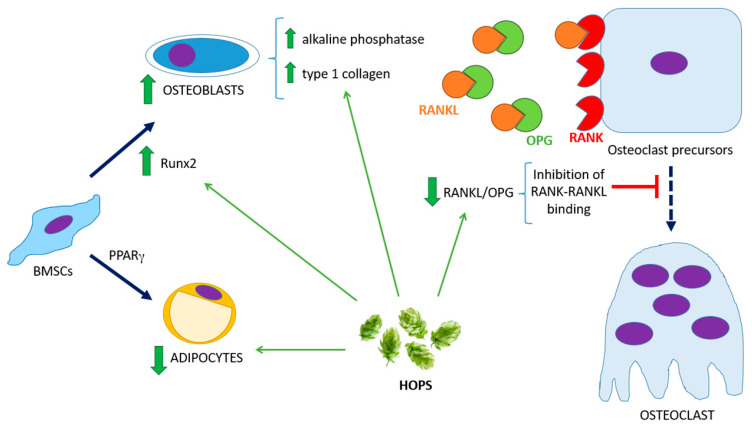
**Osteoprotective effects of hops on bone marrow mesenchymal stem cells (BMSCs) differentiation and bone metabolism.** Hops promote BMSCs’ differentiation toward the osteoblastic lineage by upregulating Runx2 expression and activating the AMPK pathway, which leads to increased alkaline phosphatase activity and type I collagen production. Furthermore, hops decreases the RANKL/OPG ratio, which inhibits osteoclast formation and, consequently, reduces bone resorption. Runx2: runt-related transcription factor 2, PPARγ: peroxisome proliferator-activated receptor gamma, RANK: receptor activator of nuclear factor κB, RANKL: receptor activator of NFκB ligand, OPG: osteoprotegerin, BMSCs: bone marrow stem cells.

Additionally, we show that oral treatment with hops extract increases the trabecular bone area and decreases bone marrow adiposity, thereby improving bone microarchitecture, in line with our *ex vivo* results with BMSCs. The higher expression of Runx2 suggests that BMSCs’ differentiation is leaning towards the osteoblastic lineage, not only promoting bone tissue synthesis but also decreasing the number of adipocytes in the bone marrow. Although we have not evaluated here the adipogenic potential of BMSCs, the reduction in bone marrow adiposity observed in the histological sections is consistent with previous studies, where we demonstrated in *in vitro* assays that hops extract decreases BMSCs’ expression of adipogenic transcription factor PPARγ [15]. Recently, several studies have investigated the effects of LPL treatment in ovariectomized rats or mice. They have shown that LPL has osteoprotective effects on bone microarchitecture by increasing bone mineral density (BMD), preventing alterations in serum levels of bone turnover markers and mitigating decreases in trabecular thickness, depending on the skeletal site [24,33,34]. Conversely, Sun and colleagues demonstrated that LPL prevented deleterious alterations in bone microarchitecture in a model of osteoporotic male mice [28]. In agreement with these findings, our results show that the beneficial effects on bone microarchitecture and bone cells are accompanied by enhanced biomechanical properties, as confirmed by three-point bending tests. Our results, summarized in Figure 3, suggest that LPL treatment not only stimulates cellular activity but also translates into structural and functional benefits for bone tissue.

Such enhancements are particularly relevant in the context of osteoporosis, where conventional treatments often present a range of side effects that deter patient adherence. The chemical composition of the hops extract presents an advantageous profile, positioning it as a viable alternative treatment option. Each of the active compounds present in the extract could act synergistically without the need for high doses of each component, resulting in a more economical and safer formulation. Our results also highlight the significance of the long-term dietary incorporation of natural compounds like hops, which may serve to prevent osteoporosis progression and improve overall skeletal health. Future studies should further investigate potential applications in clinical settings.

## 5. Conclusions

In summary, our study demonstrates that a two-month oral treatment with Hops extract significantly enhances the osteogenic potential of bone marrow stem cells and improves both bone microarchitecture and biomechanical properties in young male Sprague Dawley rats. The extract’s ability to upregulate osteogenic markers while downregulating the factors associated with bone resorption underscores its potential as a novel, natural therapeutic option for osteoporosis and other metabolic bone diseases. Given the increasing prevalence of osteoporosis and the limitations of existing pharmacological treatments, the incorporation of hops extract could offer a complementary approach to bone health management.

Clinical investigations are necessary to confirm the effectiveness of *Humulus lupulus* in dietary regimens or therapeutic protocols, designed to improve bone health and mitigate the risks associated with osteoporosis.

## Figures and Tables

**Table 1:** Primer sequences for RT-PCR of specific markers.

Marker	Genebank Code	Product Size (bp)		Sequence
*Housekeeping gene*				
**β-Actin**	NM_031144.3	**345**	**Fw**	CCTTCAACACCCCAGCCAT
			**Rv**	CATAGCTCTTCTCCAGGGA
*Markers regulating osteoblast/osteoclast differentiation*				
**Runx2** **(2 bands)**	XM_006244554.2	**598/424**	**Fw**	GCCGGGAATGATGAGAACTA
			**Rv**	TGAGAGAGGAAGGCCAGA
**RANKL**	NM_057149.1	**432**	**Fw**	TCGCTCTGTTCCTGTACTTT
			**Rv**	CCCTTAGTTTTCCGTTGCTT
**OPG**	U94330.1	**408**	**Fw**	CTCCTGGCACCTACCTAA
			**Rv**	GTGTTGCATTTCCTTTCTGA
**t1 Col**	NM_053304.1	**651**	**Fw**	GCATACACAATGGCCTAA
			**Rv**	CTGTTCCAGGCAATCCAC
**ALP**	J03572.1	**737**	**Fw**	GACAGCAAGCCCAAGAGA
			**Rv**	CAGTTCAGTGCGGTTCCA

**Table 2:** Results for femoral mechanical testing (three-point bending analysis).

Parameters	Control	LPL
Stiffness (N/mm)	237.7 ± 12.9	392.4 ± 17.3 *
Yield Point (N)	88.8 ± 3.5	120.5 ± 11.6 *
Maximum load (N)	106.4 ± 8.7	147.5 ± 13.2 *
Work to fracture (T-Eabs, Nmm)	18.4 ± 1.9	31.1 ± 4.0 *
P-Eabs (Nmm)	15.4 ± 1.2	24.2 ± 3.4
E-Eabs (Nmm)	3.0 ± 1.0	6.9 ± 1.9

Results are expressed as the mean ± SEM. Differences: * *p* < 0.05 vs. control. For each parameter, the number of femora evaluated was n = 5.

## Data Availability

All the data in the article are available from the corresponding author upon reasonable request.

## Abbreviations

6-PN	6-prenylnaringenin
8-PN	8-prenylnaringenin
ALP	alkaline phosphatase activity
BMSCs	bone marrow stem cells
C	control group
DMEM	Dulbecco’s Modified Eagle Medium
Dy	displacement
Eabs	absorbed energy
E-Eabs	elastic absorbed energy
ER-α	estrogen receptor α
FBS	fetal bovine serum
Fmax	maximum load
Fy	yield load
H&E	hematoxylin–eosin
IGF-1	insulin-like growth factor 1
IL-1	interleukin-1
IL-6	interleukin-6
IXan	isoxanthohumol
LPL	hop extract
MMLV-RT	Moloney Murine Leukemia Virus Reverse Transcriptase
Oc	osteocalcin
OP	osteoporosis
OPG	osteoprotegerin
PBS	phosphate-buffered saline
P-Eabs	plastic absorbed energy
p-NP	p-nitrophenol
p-NPP	p-nitrophenyl phosphate
RANK	receptor activator of nuclear factor κB
RANKL	receptor activator of NFκB ligand
RT-PCR	reverse transcriptase polymerase chain reaction
Runx2	Runt-related transcription factor 2
T-Eabs	total absorbed energy
TGF-β	transforming growth factor-beta
TNF-α	tumor necrosis factor-alpha
Xan	xanthohumol
